# Supplementary material for: Self-organization of swimmers drives long-range fluid transport in bacterial colonies
Source: Nat Commun. 2019 Apr 17;10:1792. doi: 10.1038/s41467-019-09818-2 (PMC6470179; doi:10.1038/s41467-019-09818-2)
Supplement: Supplementary file 1 — Supplementary Information [file 41467_2019_9818_MOESM1_ESM.pdf]

Supplementary information for

Self-organization of swimmers drives long-range fluid transport in bacterial colonies

Xu *et al*.

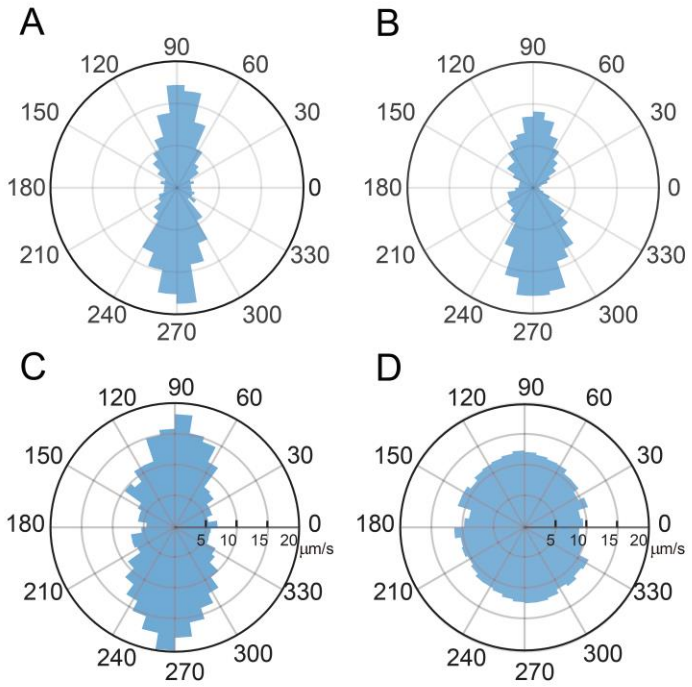

Supplementary Figure 1. (A,B) Angular probability distribution of single-cell velocity direction in the inner motile ring of a naturally grown *P. mirabilis* colony (panel A) and of a *P. mirabilis* suspension drop (panel B), respectively. The velocity direction is represented by angles ranging from 0° to 360°. 90° and 270° correspond to +Y (clockwise along the edge) and -Y (counterclockwise along the edge) directions in the coordinate system specified in main text Fig. 1B and Fig. 2A, respectively. The radii of colored circular sectors represent probability density. The probability distribution of cells' moving direction in the inner motile ring is centered around 90° and 270°, i.e. preferentially parallel to the boundary bi-directionally. (C,D) Directional dependence of average speed in the inner motile ring of a naturally grown *P. mirabilis* colony (panel C) and of a *P. mirabilis* suspension drop (panel D), respectively. The radii of colored circular sectors represent the magnitude of speed, with the scale indicated in the plots. Note that the directional dependence of average speed is more isotropic in suspension drops than that in natural colonies; this is because there is no sessile part in suspension drops and thus cells are able to move between the inner motile ring and the dilute phase in all directions without much obstruction (or reduction of speed).

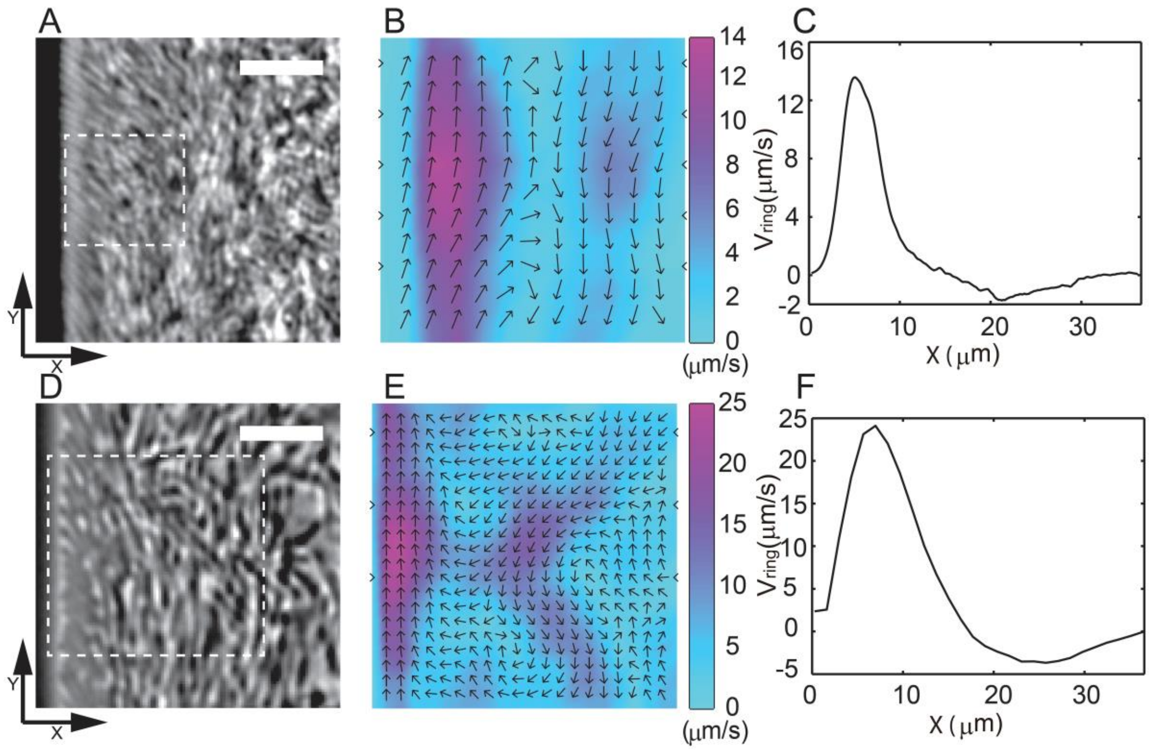

Supplementary Figure 2. Self-organization of two adjacent colony-scale motile rings at the edge of *E. coli* and *B. subtilis* colonies. (A, D) Phase contrast image of *E. coli* (A) and *B. subtilis* (D) colony edge. Scale bar, 20μm. Also see Supplementary Movie 3,4. (B, E) Time-averaged collective velocity field of cells in the selected region of panel A or D (enclosed by dashed rectangle) computed by Horn–Schunck analysis (B; associated with panel A) or particle image velocimetry (PIV) analysis (E; associated with panel D) based on phase contrast images. PIV analysis was performed using an open-source package MatPIV 1.6.1 written by J. Kristian Sveen, http://folk.uio.no/jks/matpiv/index2.html). For each pair of consecutive images, the interrogation-window size started at 10.4 μm x 10.4 μm and ended at 5.2 μm × 5.2 μm after 4 iterations. The grid size of the resulting velocity field was 2.6 μm × 2.6 μm. The collective velocity field was averaged over a duration of 10 s. Arrows represent velocity direction, and colormap represents velocity magnitude (with the color bar provided to the right, in μm s^{-1}). (C, F) The mean tangential speed of collective cellular motion (C: based on optical flow data; F: based on PIV data) plotted against the distance from colony edge. In both panels C&F, positive value of speed indicates motion along CW direction, i.e. along +Y axis in the coordinate system specified in panel A or D, and X = 0 is set at the position of colony edge.

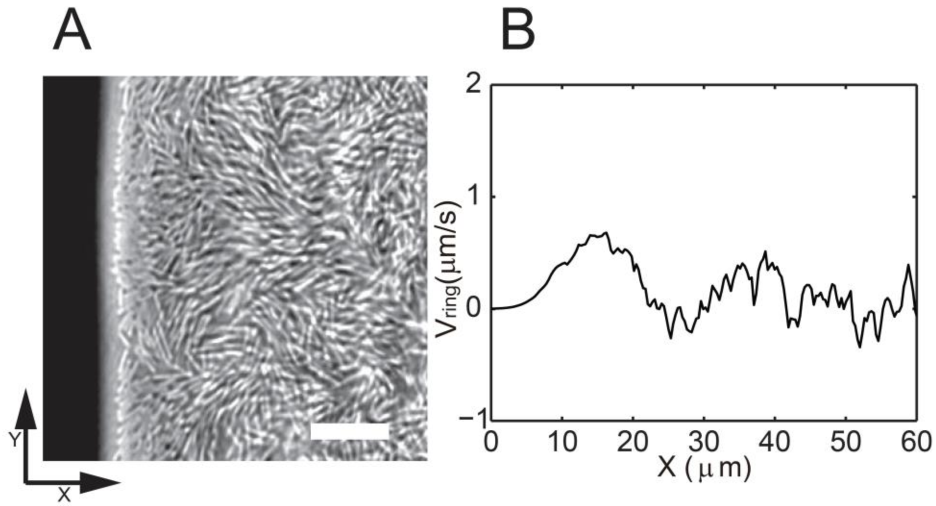

Supplementary Figure 3. Motion pattern of smooth swimming *B. subtilis* at colony edge. (A) Phase contrast image of the colony edge of smooth swimming *B. subtilis* mutant (DK2178). Scale bar, 20 μm. (B) The mean tangential speed of smooth swimming *B. subtilis* cells (computed by optical flow analysis with phase contrast images) is plotted against the distance from colony edge. Positive value of speed indicates motion along CW direction, i.e. along +Y axis in the coordinate system specified in panel A, and X = 0 is set at the position of colony edge. Also see Supplementary Movie 5.

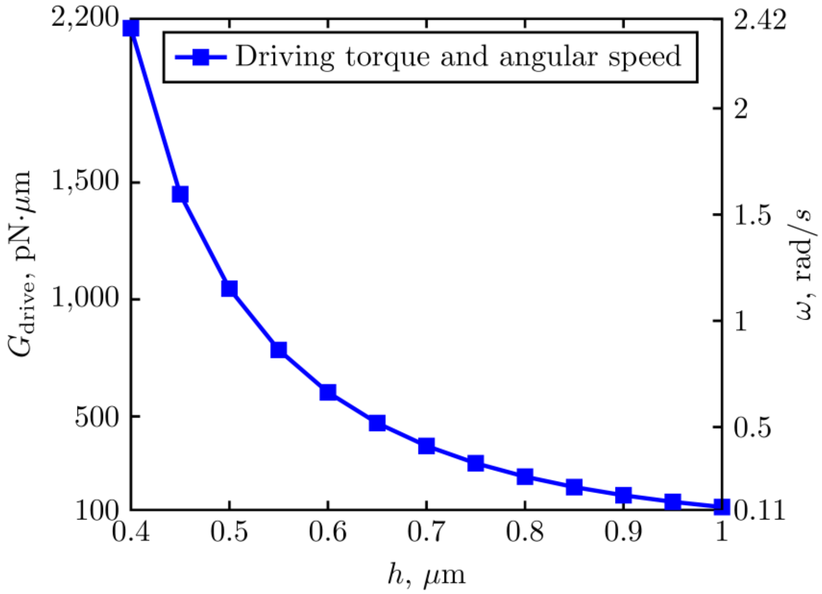

Supplementary Figure 4. Results of theoretical model for cell reorientation during collision with colony edge. The driving reorientation torque G_{drive} about the front end of the cell (which is in contact with the drop edge) and the reorientation angular speed shown in Fig. 3F are plotted as a function of the flagellar axis height *h* above the substrate.

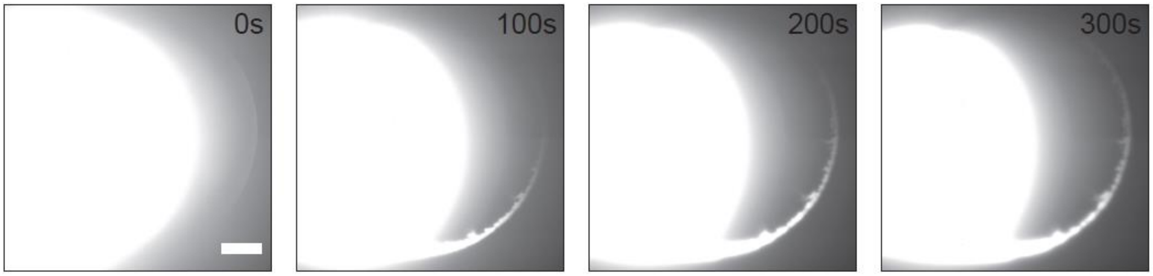

Supplementary Figure 5. Long-range, colony-scale directed transport in a *P. mirabilis* colony revealed by FITC–Dextran (Methods). Bright blob in the left part of the images is the deposited dextran solution. Scale bar, 500 μm.

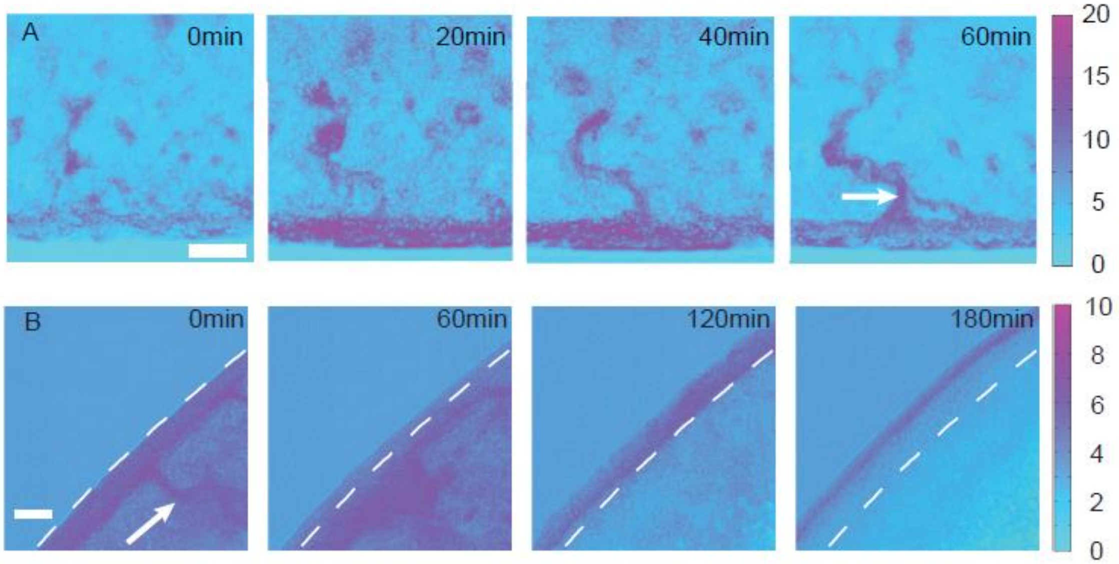

Supplementary Figure 6. Formation and disappearance of cracks near *P. mirabilis* colony edge. (A) Image sequence showing the time-averaged collective velocity field of cells during crack formation (computed by optical flow analysis). The arrow in the last image indicates a fully developed crack. (B) Image sequence showing the time-averaged collective velocity field of cells during crack disappearance (computed by optical flow analysis). The arrow in the first image indicates the position of a crack, and the dashed lines indicate the position of colony edge in the first image. Note that, as the lifetime of cracks spans many hours and we found that the cracks are prone to environmental perturbation, crack formation (panel A) and disappearance (panel B) were imaged separately. Scale bars, 50 μm. T=0 in panel A and B corresponds to 15 hr and 48 hr after inoculation, respectively.

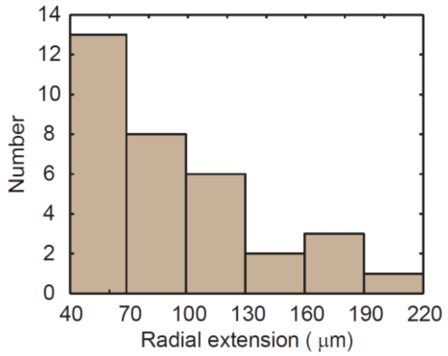

Supplementary Figure 7. Distribution of radial extension of cracks. Cracks were visualized by FITC–Dextran at 24 hr after inoculation (Methods). The data are based on 33 cracks visualized in 20 colonies, and only the cracks with a radial extension greater than 40 μm are considered.

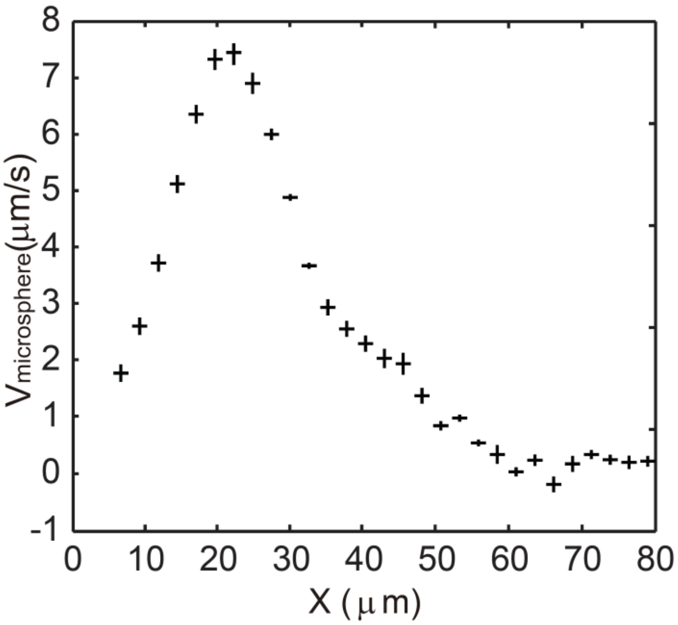

Supplementary Figure 8. Mean tangential flow speed plotted against the distance from the edge of a *P. mirabilis* suspension drop. Fluid flows were visualized by 0.1 μm diameter microspheres and the flow speed was measured by tracking single microspheres. To avoid the influence of cells in the inner motile ring on microsphere motion (as microspheres tend to get stuck to cells), flow visualization was done when only the CW outer motile ring appeared at the edge and the inner motile ring had not developed yet. Positive value of speed indicates motion along CCW direction, and X = 0 is set at the position of the edge of the suspension drop. About 3000 microspheres were tracked in ~5-min movie and their trajectories were divided into ~ 20000 segments each with a duration of 1 s. The X-axis was binned, and the tangential component of the mean velocity of those 1-s segments falling into each bin was computed to yield data in this plot. Horizontal error bars indicate the bin width for the computation of mean tangential speed; vertical error bars indicate standard error of the mean (n= number of 1-s segments falling into the bin; >100).

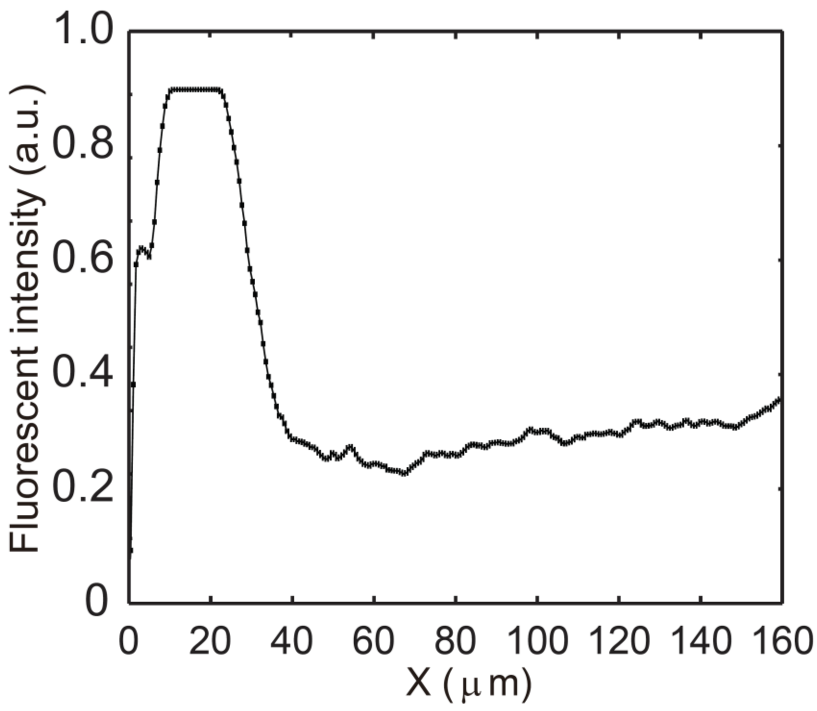

Supplementary Figure 9. Surface cell density plotted against the distance from the edge of a *P. mirabilis* suspension drop. The surface cell density (number of cells per unit area of substrate surface) is represented by fluorescence intensity, as all cells in the suspension drop were fluorescently labeled (expressing GFP). Fluorescence intensity of cells was measured when the CW outer motile ring and CCW inner motile ring both appeared and had stabilized at the edge.

A

The effect of LB agar concentration on the growth of *P. mirabilis* colonies with relative humidity 85%RH

	LB concentration (%)			
	0.6	1.0	1.5	2.0
Sessile colony with motile rings	Yes	Yes	Yes	Yes
Initiation of motile rings (hour)	19 ± 1	19 ± 1	17 ± 1	17.5 ± 0.5
Swarming	Yes	Yes	Yes	Yes
Initiation of swarming (hour)	> 24	> 24	> 24	> 24

B

The effect of relative humidity on the growth of *P. mirabilis* colonies with LB agar concentration 0.6%.

	Relative humidity (%RH)					
	97.0	85.0	60.4	55.0	47.4	42.0
Sessile colony with motile rings	Yes	Yes	Yes	Yes	n.a.	n.a.
Initiation of motile rings (hour)	19 ± 1	19 ± 1	15.5 ± 0.5	14.5 ± 0.5	n.a.	n.a.
Swarming	Yes	Yes	Yes	Yes	Yes	Yes
Initiation of swarming (hour)	> 24	> 24	> 24	> 24	4.5 ± 0.5	4.5 ± 0.5

Supplementary Table 1. Environmental effect on motile-ring formation. (A) The effect of LB agar concentration on the growth of *P. mirabilis* colonies cultured at 85% relative humidity. (B) The effect of relative humidity on the growth of *P. mirabilis* colonies cultured on 0.6% LB agar. The ± sign indicates uncertainty in time measurement (n=8 biologically independent samples for each data).
