# Supplementary material for: Self-organization of swimmers drives long-range fluid transport in bacterial colonies
Source: Nat Commun. 2019 Apr 17;10:1792. doi: 10.1038/s41467-019-09818-2 (PMC6470179; doi:10.1038/s41467-019-09818-2)
Supplement: Supplementary file 3 — Description of Additional Supplementary Files [file 41467_2019_9818_MOESM3_ESM.pdf]

Description of Additional Supplementary Files

File Name: Supplementary Movie 1
Description: Self-organization of two adjacent colony-scale motile rings at the edge of a *P. mirabilis* colony. This phase-contrast video is played at 10 frames per s with the real elapsed time indicated in the time stamp. It is associated with Fig. 1(A-D) in main text. Scale bar, 20µm.

File Name: Supplementary Movie 2
Description: Single cell motion pattern at the edge of a *P. mirabilis* colony. This video was obtained via fluorescence imaging. Fluorescently labeled cells were mixed with wildtype cells at a ratio of 1:2000. The video is played at 5 frames per s with the real elapsed time indicated in the time stamp. It is associated with Fig. 1(E) in main text. Scale bar, 20µm.

File Name: Supplementary Movie 3
Description: Self-organization of two adjacent colony-scale motile rings at the edge of an *E. coli* colony. This phase-contrast video is played at 30 frames per s with the real elapsed time indicated in the time stamp. Scale bar, 10µm.

File Name: Supplementary Movie 4
Description: Self-organization of two adjacent colony-scale motile rings at the edge of a *B. subtilis* colony. This phase-contrast video is played at 30 frames per s with the real elapsed time indicated in the time stamp. Scale bar, 20µm.

File Name: Supplementary Movie 5
Description: Motion pattern of smooth swimming *B. subtilis* at colony edge. This phase-contrast video is played at 30 frames per s with the real elapsed time indicated in the time stamp. Scale bar, 20µm.

File Name: Supplementary Movie 6
Description: Self-organization of two adjacent motile rings at the edge of *P. mirabilis* suspension drop (artificial colony). This phase-contrast video is played at 20 frames per s with the real elapsed time indicated in the time stamp. It is associated with Fig. 2A in main text. Scale bar: 20 µm.

File Name: Supplementary Movie 7
Description: Self-organization of two adjacent motile rings at the edge of *P. mirabilis* suspension drop (artificial colony). This video was obtained via fluorescence imaging and all cells in the colony were GFP-tagged. It is played at 30 frames per s with the real elapsed time indicated in the time stamp. This video is associated with Fig. 2A in main text. Scale bar: 20 µm.

File Name: Supplementary Movie 8
Description: Single cell motion pattern near the edge of a diluted *P. mirabilis* colony. This phase-contrast video is played at 5 frames per s with the real elapsed time indicated in the time stamp. It is associated with Fig. 3(A-B) in main text. Black dashed trace indicates the trajectory of a chosen cell. Scale bar, 20 µm.

File Name: Supplementary Movie 9
Description: Single cell motion pattern near the edge of a low-density *E. coli* suspension drop. This video was obtained via fluorescence imaging. Cells were GFP-tagged and their flagellar filaments were fluorescently labeled (Methods). The video is played at 20 frames per s with the real elapsed time indicated in the time stamp. White dashed line indicates the edge of suspension drop. Scale bar, 20 µm.

File Name: Supplementary Movie 10
Description: Single cell motion pattern near the edge of a low-density *B. subtilis* suspension drop. This phase-contrast video is played at 5 frames per s with the real elapsed time indicated in the time stamp. Scale bar, 10 µm.

File Name: Supplementary Movie 11
Description: Long-range, colony-scale directed transport of microspheres in a *P. mirabilis* colony. Microspheres were imaged in fluorescence and the colony was imaged in phase-contrast (at low light intensity) (Methods). This video is played at 10 frames per s with the real elapsed time indicated in the time stamp. It is associated with Fig. 4(A-D) in main text. Scale bar, 500 µm.

File Name: Supplementary Movie 12
Description: Directed transport of fluorescent microspheres inwards along crack-like conduits present near the edge of a *P. mirabilis* colony. Microspheres were imaged in fluorescence and the colony was imaged via phase-contrast (Methods). This video is played at 20 frames per s with the real elapsed time indicated in the time stamp. It is associated with Fig. 4(E-H) in main text. Scale bar: 50 µm.
